# Laser Navigation Combined With XperCT Technology Assisted Puncture of Brainstem Hemorrhage

**DOI:** 10.3389/fneur.2022.905477

**Published:** 2022-06-09

**Authors:** Qingbo Wang, Wei Guo, Tao Zhang, Shuangquan Wang, Chenglong Li, Zhengbo Yuan, Qi Wei, Xin Geng, Zefu Li

**Affiliations:** ^1^Department of Neurosurgery, Qilu Hospital of Shandong University, Jinan, China; ^2^Department of Neurosurgery, Binzhou Medical University Hospital, Binzhou, China; ^3^Department of Neurology, Binzhou Medical University Hospital, Binzhou, China

**Keywords:** brainstem hemorrhage, hematoma puncture drainage, laser navigation, XperCT technology, individualized and precision medicine

## Abstract

**Background:**

Brainstem hemorrhage has a rapid onset with high mortality and disability rates. In recent years, an increasing number of studies have reported on the surgical treatment of brainstem hemorrhage. The introduction of stereotaxic instruments and navigation systems has improved the accuracy of surgical treatment; however, the popularity of these devices in the primary hospitals is not high. In this study, we introduce laser navigation combined with the XperCT technology to assist in the puncture and drainage of brainstem hemorrhage, aiming to improve surgical accuracy and facilitate the drainage of brainstem hemorrhage in primary hospitals.

**Material and Methods:**

A total of five patients (four men and one woman), aged 34–70 years, who underwent hematoma puncture drainage with the assistance of laser navigation combined with XperCT technology at the Binzhou Medical University Hospital, China, between June 2020 and Aug 2021 were included in the study. The brainstem hemorrhages had volumes of 7–18 ml. Statistical analyses of the postoperative puncture deviation distance (distance between the actual puncture end and simulated puncture end) and postoperative improvement were also performed.

**Results:**

The operations were successfully completed in all five patients. The puncture deviation distance was <6 mm in all five patients and <2 mm in two patients. The postoperative hematoma clearance rate was about 70%−90%. Among four patients with respiratory failure, three had improved breathing and resumed spontaneous breathing. Out of three patients with high fever, one showed a substantial decrease in body temperature. There were no cases of postoperative infection. Of the five patients, two recovered consciousness, one died, and two voluntarily gave up further treatment and were discharged.

**Conclusions:**

Laser navigation combined with the XperCT technology could improve the accuracy of surgical puncture. The technique might be convenient for widespread clinical application because of its low trauma, high precision, short operation time, and low operation cost.

## Introduction

Brainstem hemorrhage, which accounts for about 10% of spontaneous intracerebral hemorrhage, typically presents with acute onset, severe symptoms, and high disability and mortality (1, 2). The etiologies of brainstem hemorrhage include hypertension, vascular malformation, tumor stroke, and coagulation dysfunction. The clinical manifestations can be classified into two main categories: (1) consciousness disorder syndrome caused by damage to the midbrain and pons; (2) vital sign disorder syndrome caused by damage to the medulla (3, 4). The pathological damage in brainstem hemorrhage includes damage to the reticular formation and regions controlling respiration and heartbeat, resulting in coma, respiratory failure, and unstable blood pressure, which may pose a serious life threatening. After hemorrhage, a hematoma typically forms, which squeezes, deforms, displaces, or even tears the brainstem tissue (5, 6), causing severe ischemia and hypoxic edema of the brainstem, and eventually leading to further damage to vital centers (7). Depending on the location of the bleeding, the blood mass may flow into and compress the ventricles, which can impede the circulation of cerebrospinal fluid, thus deepening the patient's coma. In addition, bloody inflammatory stimulators continue to stimulate the brainstem tissue after hemorrhage, resulting in complications such as high fever, further increasing mortality rates (8–10). Early and effective removal of hematoma, as well as relieving its pressure and hemorrhagic toxicity on brain stem tissue (5), may be essential in improving the patient's condition.

At present, the treatment for brainstem hemorrhage is mainly conservative (3, 11). In this study, we described our use of laser navigation combined with XperCT technology to perform puncture and drainage of brainstem hemorrhage in five patients, with the aim of improving the accuracy of surgery and obtaining clinical efficacy.

## Materials and Methods

### General Information

The present study cohort included five patients, four men and one woman, aged 34–70 years old, who underwent laser navigation combined with XperCT technology for assisted hematoma puncture drainage at the Binzhou Medical University Hospital, People's Republic of China, between June 2020 and Aug 2021. The procedure was reviewed and approved by the Ethics Committee of the Binzhou Medical University Hospital (KT-062). Consent for the operations was obtained from each patient's family. The volume of brainstem hemorrhage ranged from 7 to 18 ml. All five patients had hypertension and were in a deep comatose state in the early stage of the disease. About four patients had respiratory failure, and three had a high fever. The interval time from onset to surgery in five patients ranged from 6 to 26 h (Table 1).

**Table 1:** The basic information of patients.

**Case No**.	**Age (years), sex**	**GCS (pre-operation)**	**Respiratory failure (Y or N)**	**Hyperpyrexia (Y or N)**	**Bleeding volume (ml)**	**How soon begin to surgery after the onset (h)**
1	70, M	4	N	N	9	6
2	67, F	7	Y	N	7	15
3	57, M	5	Y	Y	8	8
4	34, M	3	Y	Y	18	26
5	58, M	4	Y	Y	12	25

### Indications and Contraindications for Surgery

The indications for surgery were: (1) relatively concentrated hematoma with a volume ≥5 ml or transverse hematoma diameter >2 cm; (2) GCS score <8 points accompanied by progressive neurological dysfunction; (3) unstable vital signs, such as ventilator-assisted breathing and central high fever.

The contraindications for surgery included: (1) non-primary brainstem hemorrhage; (2) unstable blood pressure and heart rate; (3) severe heart, lung, liver, and kidney disease; (4) coagulation dysfunction.

### Equipment

**Figure 1:**
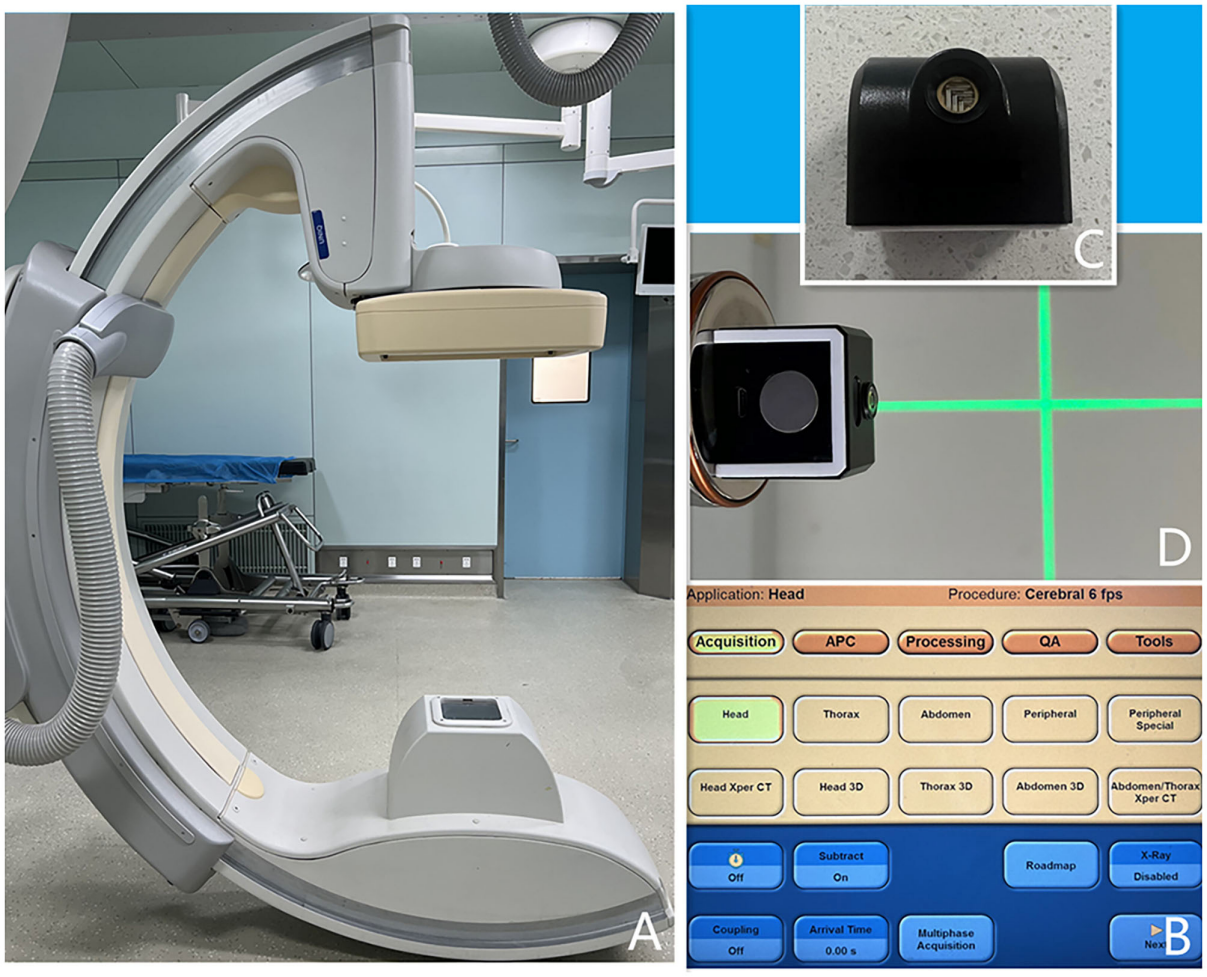
**(A)** XperCT equipment. **(B)** Software operation interface. **(C,D)** Laser locator.

Laser locator (ZhonNa NX-9575-675, made by ZhonNa Electronics Company Of Zhongshan), XperCT equipment(UNIQ FD20, made by Philips Medical Systems Nederland B.V.) (Figure 1).

### Procedural Methods

#### Determination of Percutaneous Puncture Points

**Figure 2:**
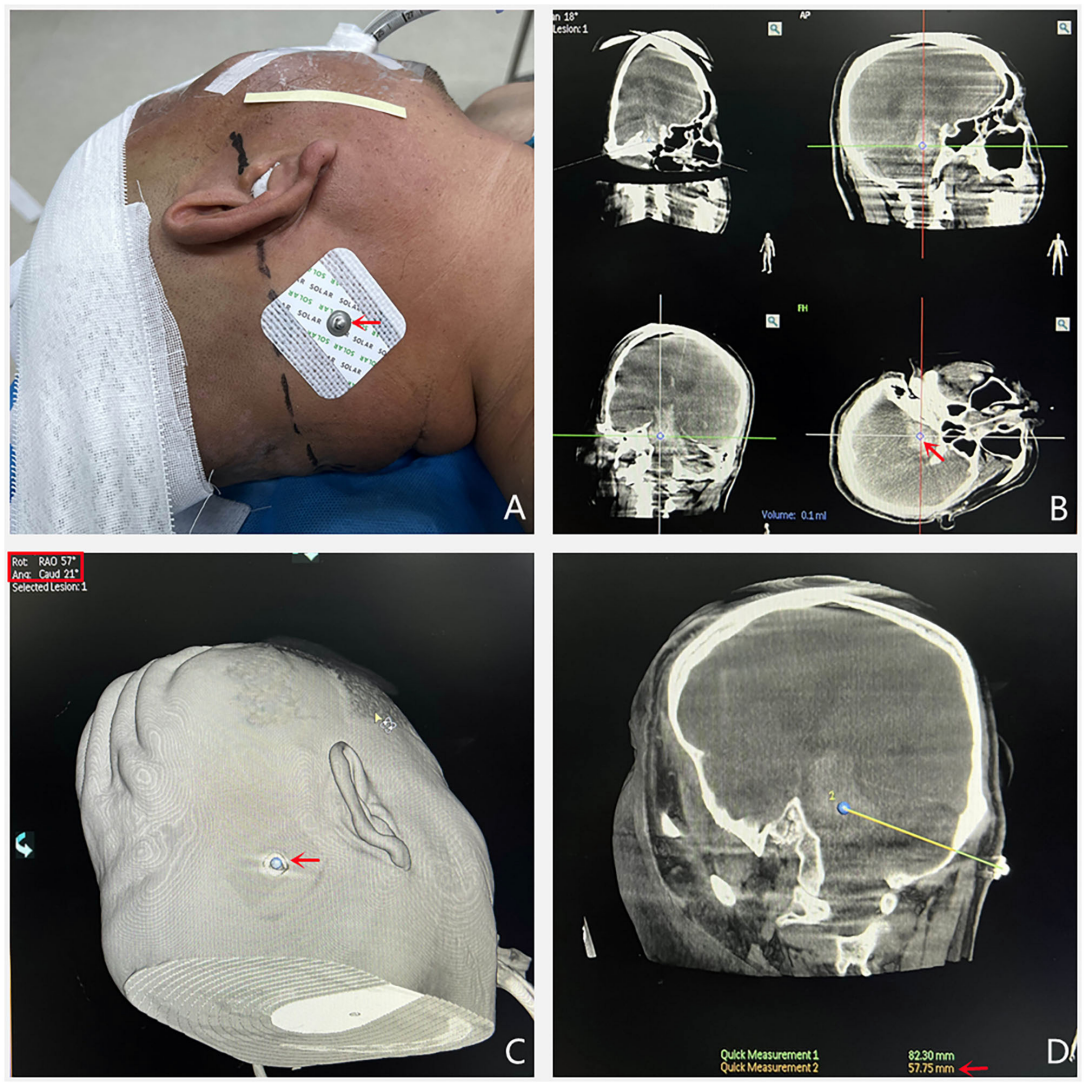
**(A)** The percutaneous puncture point was marked with electrode pastes. **(B)** XperCT examination was performed and the center of the largest layer of hematoma was identified as the puncture target. **(C)** When the percutaneous puncture point and the puncture target are overlapped, the software will display two angles. **(D)** The puncture direction is the line between the percutaneous puncture point and puncture target. The puncture depth is the distance between the puncture target and the cortical brain tissue.

The area 2–3 cm behind the mastoid and 1.5–2 cm below the transverse sinus was selected as the areas of percutaneous puncture point, which were marked with electrode paste (which is easily visible on CT) (Figure 2A). The puncture points were selected so as to avoid the sinus and important functional areas.

#### XperCT Scan

The patient was placed in the lateral position, the posterior occiput of the puncture side was exposed, and the head was fixed, after which the patient underwent the XperCT scan.

#### Determination of Puncture Target

Software supplied with the machine was used for 3D reconstruction to determine the maximum axial, coronal, and sagittal levels of the hematoma, and the central point of the maximum level of the hematoma was determined as the puncture target (Figure 2B).

#### Measurement of Puncture Depth

The measured distance between the percutaneous puncture point and the puncture target was taken as the actual intraoperative puncture depth (Figure 2D).

#### Determination of the Puncture Direction

**Figure 3:**
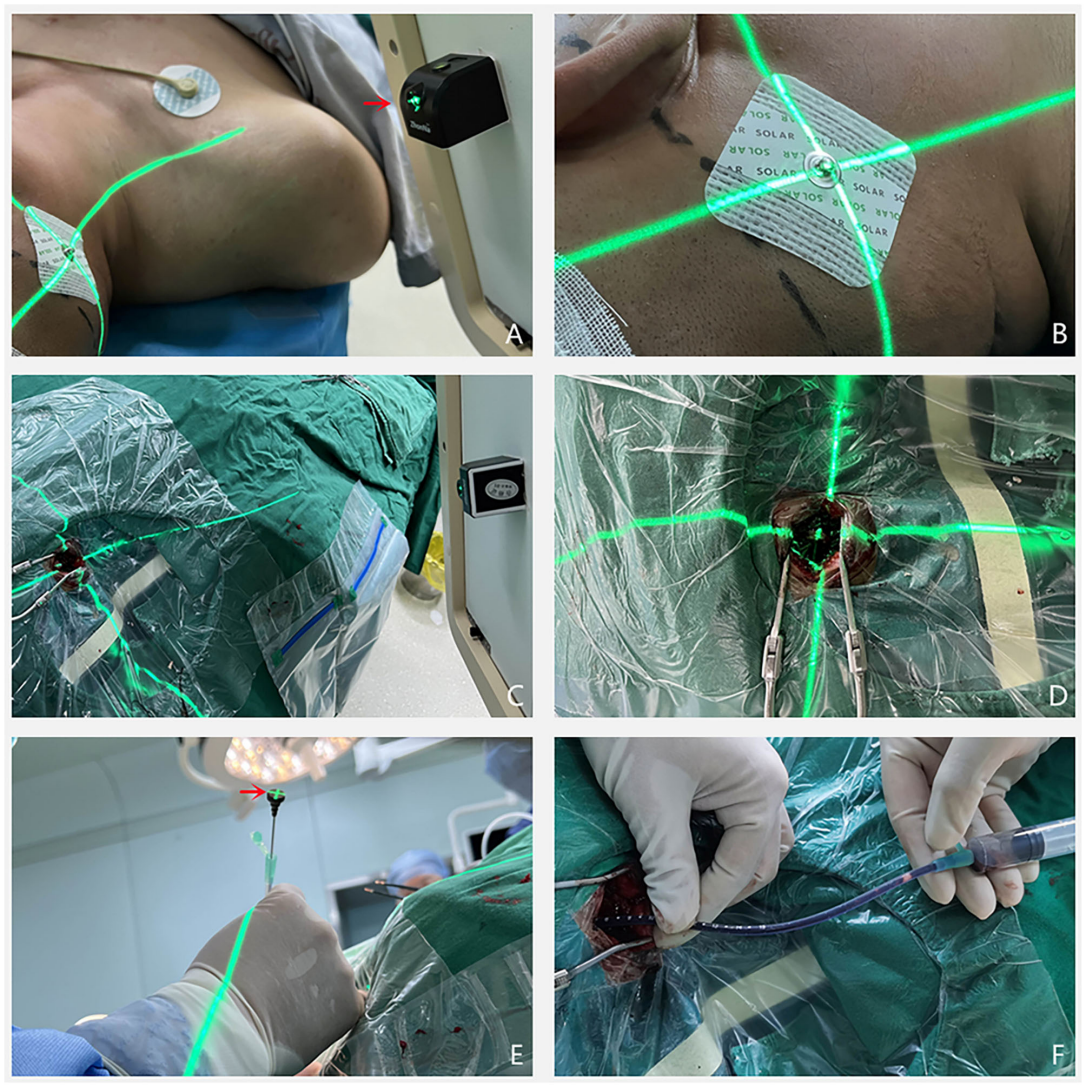
**(A,B)** The laser transmitter (pointed by the red arrow) was placed on the C-shaped arm and adjusted at a predetermined angle, so that the center of the laser points to the percutaneous puncture point, and the direction of laser irradiation is the puncture direction. **(C–E)** The scalp was incised with the percutaneous puncture point as the center, and the cortical brain tissue was exposed in the direction of laser emission. The tip of the punctured tube pointed to the intersection of the laser and the cortical brain tissue, and the end of the punctured tube always coincided with the center of the laser. **(F)** The needle was punctured to a predetermined depth, after which a 5 ml syringe was used to slowly aspirate a dark red hematoma to confirm that the drainage tube was located in the hematoma cavity.

The percutaneous puncture point and puncture target were overlaid (two points were connected). At this point, the angle displayed by the machine is the spatial angle of the puncture direction, and the C-arm direction is adjusted to this angle (Figure 2C). The laser locator is fixed on the C-arm plate and the laser projection position (the cross point) is fixed to the percutaneous puncture point, at which point the laser direction is the puncture direction (Figures 3A,B).

#### Surgery

After the patient was anesthetized, a straight skin incision of about 3 cm in length was made with the percutaneous puncture point as the center, and a bone hole was drilled. After the dura was incised, the tip of the puncture and drainage tube was passed through the percutaneous puncture point while ensuring the crossing of the laser (Figures 3C,D). The point always pointed to the center of the external end of the puncture needle (Figure 3E), and the drainage tube was slowly advanced and pushed into place according to the pre-measured puncture depth. When dark-red bloody fluid flew out, or the syringe slowly drew a dark-red blood clot (Figure 3F), the drainage tube entered the hematoma cavity and was fixed. A urokinase injection could be of assistance, according to postoperative CT and hematoma drainage.

## Results

The operation was successfully completed in all five patients, with no intraoperative deaths and slight fluctuation in the patient's vital signs. The operation times are shown in Table 2.

**Table 2:** Surgical information and postoperative patient outcomes.

**Case No**.	**Operation duration (min)**	**Offset distance (mm)**	**Penetration depth (cm)**	**Respiration improved (Y or N)**	**Temperature down (Y or N)**	**Outcome**
1	41	5	6	/	/	Abandon
2	38	2	7	Y	/	Recover consicousness
3	46	4	6	Y	Y	Recover consicousness
4	36	3	8	Y	N	Abandon
5	32	2	6.5	/	N	Death

**Figure 4:**
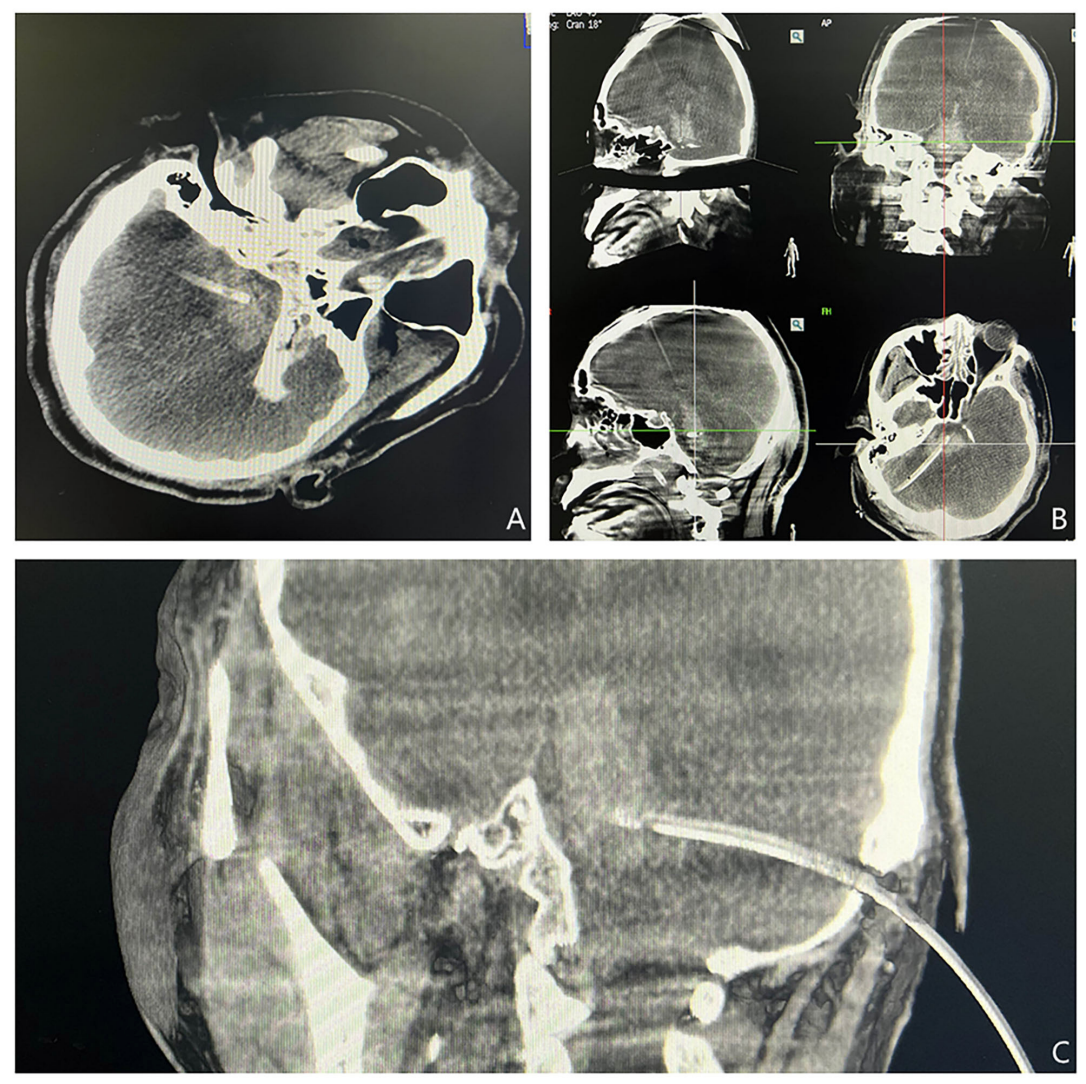
**(A–C)** XperCT was performed again to determine the location of the drainage tube. The positions of the drainage tubes shown in the axial, coronal, and sagittal views were satisfactory.

The postoperative CT results showed that the end of the punctured tube was in the hematoma cavity in all five cases (Figure 4). The distance between the actual puncture end and the simulated puncture end, also referred to as the deviation distance, was calculated from the 3D reconstruction. The deviation distance was ≤ 6 mm in all five patients, and <2 mm in two of the patients.

The hematoma drainage was satisfactory after being combined with a urokinase injection. Generally, the catheter was extubated 3–5 days after the operation, and the hematoma clearance rate was about 70%−90%. Among the four patients with respiratory failure, three had improved breathing and resumed spontaneous breathing. One of the three patients with high fever showed a significant decrease in body temperature following treatment. There were no cases of postoperative infection. Among the five patients, two recovered consciousness, one died, and two voluntarily gave up further treatment and were discharged (Table 2).

## Discussion

The brainstem is composed of three parts: the midbrain, pons, and medulla oblongata. It is the circulatory and respiratory center of the human body, and also the link between the central nervous system and the peripheral nervous system (12, 13). In the early stage of brainstem hemorrhage, severe clinical manifestations such as deep coma, high fever, and breathing disorder can occur, as the hemorrhage causes damage to the brainstem tissue, such as the reticular activation system and the medulla oblongata respiratory center (14), and eventually high disability and fatality rates. At present, the treatment for brainstem hemorrhage is mostly conservative, such as maintaining the stability of vital signs and correcting water and electrolyte imbalances. Because of different cultures between Eastern and Western countries, the guidelines for treating cerebral hemorrhage formulated by European countries and the United States do not explicitly mention the surgical treatment of brainstem hemorrhage (3, 11). They believe that the overall prognosis for brainstem hemorrhage is poor, and that severe postoperative disability or a vegetative state may have important consequences for society and family. Because of extremely high economic and mental burdens, surgery is not recommended. There have been many studies on the surgical treatment of brainstem hemorrhage in Asia in recent years. Ichimura reported promising results for the surgical treatment of patients with brain stem hemorrhage (15). In South Korea, Hong et al. performed craniotomy on 13 patients with severe pontine hemorrhage, finding that surgical operation within 24 h of onset was ineffective, whereas surgical operation 6–20 days after onset was effective for some patients whose condition worsened during conservative medical treatment (16). Therefore, microscopic craniotomy for hematoma removal can be considered an alternative treatment for primary brainstem hemorrhage. In China, reports on the surgical treatment of brainstem hemorrhage have gradually increased, and the prognosis in certain conditions has improved over recent years (7, 17, 18). Although surgical treatment cannot reverse the primary damage, the early release of hematoma compression and hemorrhagic inflammatory stimulation can effectively reduce the secondary damage and improve the prognosis of patients. Most of the vital signs are unstable for patients with severe brainstem hemorrhage, and they cannot tolerate prolonged craniotomy. With the development of stereotaxic technology, stereotaxic hematoma aspiration has gradually become the main treatment method for brainstem hemorrhage because it is minimally invasive, precise, and less time-consuming. However, stereotaxic technology requires fixing the patient's head in a frame, thus causing additional pain to the patient. In addition, the stereotaxic apparatus is expensive and is not convenient for application in primary hospitals. Our previous study used a 3D printed guide plate to assist the puncture of brainstem hematoma, achieving certain results. The puncture catheter could reach the hematoma cavity smoothly with the help of the guide plate; however, but the design, printing, and disinfection of the guide plate took time, which limited its potential application.

After understanding the advantages and disadvantages of stereotaxic technology and 3D printing technology in the treatment of severe brainstem hemorrhage, we introduced a new technology for minimally invasive treatment of brainstem hemorrhage, i.e., laser combined with XperCT technology, which was inspired by the hybrid operating room digital subtraction angiography machines, employing the super-selection capabilities of imaging machines. In the case of the over-selection function, we found that the upper left corner of the software displayed two angles, which reminded us of the arc angle and ring angle of the stereographer. A plane can be determined with these two angles, which can be set perpendicular to the puncture direction, and after which the linear stability of the laser can guide the puncture. All five patients in this study presented with severe brainstem hemorrhage. After excluding hemangioma and vascular malformation, laser combined with XperCT technology was used to assist brainstem hematoma puncture and drainage. Before surgery, the patients' conditions were fully communicated to the families. Western countries may adopt more conservative treatment methods for patients with brainstem hemorrhage. In the Chinese culture, the survival of patients is the spiritual pillar of a family, and it is therefore meaningful to take relatively active treatment methods. However, while minimally invasive and economical methods do not bring a great burden to the patient's family, the early release of the hematoma compression may improve the prognosis of the patient. In this study, the surgical treatment was successfully completed in all five patients, and no intraoperative death occurred. Postoperative re-examination CT showed that the drainage tubes were all located in the hematoma cavity. Measurements showed that the deviation between the end position of the drainage tube and the planned target was within 6 mm in all five patients, and within 2 mm in two patients. The hematoma clearance rate was ~75%−90% after 3–5 days of the operation. Among the four patients with respiratory failure, three had improved breathing and resumed spontaneous breathing without a ventilator. Also, one of three patients with high fever showed a substantial decrease in body temperature. Among the five patients, two regained consciousness, one died, and the other two (with a hospitalization time of <3 days) voluntarily gave up further treatment and were discharged (Table 2). The postoperative prognostic outcomes of these patients increased our confidence in surgically treating patients with brainstem hemorrhage. Active surgical intervention with the early release of hematoma compression and toxicity can improve the patient's vital signs and even their state of consciousness.

Laser combined with the XperCT technology for assisting brain stem hematoma puncture and drainage has the following advantages: (1) small surgical trauma. Compared with hematoma removal through craniotomy, hematoma puncture reduces the traction damage to brain tissue and preserves nerve function as much as possible. Craniotomy for hematoma removal requires adequate exposure to the hematoma, resulting in cerebral vascular and nerve damage. In addition, the longer craniotomy operation time increases the risk of anesthesia and the probability of surgical infection. The preoperative planning of this technology can effectively avoid important blood vessels and nerves, and the operation time is short, which greatly reduces surgical trauma compared with craniotomy. (2) Application implementation is simpler and faster. Compared with the stereotaxic technique, head frame fixation is not required, which reduces the pain as well as preoperative preparation time. Compared with 3D printing technology, there is no need to produce mask guides, which saves preoperative preparation time. This technique does not require any special preparation before surgery, and the surgery can be performed after satisfactory anesthesia. (3) The operation is easy to popularize and apply. Only a digital subtraction angiography machine and a laser transmitter are needed to complete the operation. The operation process is simple, and neurosurgeons can quickly master the technology in a short time after simple training. (4) It can serve the purpose of real-time navigation. The use of XperCT technology can effectively avoid important structures and blood vessels. The position of the drainage tube can be adjusted according to the scan results in time to minimize puncture damage, thereby truly achieving the purpose of precision medicine. Conservative treatment of brainstem hemorrhage is mainly symptomatic and supportive treatment such as maintaining stable vital signs, dehydration, and lowering intracranial pressure. Hematoma absorption occurs relatively slow, and persistent hematoma compression of brainstem tissue tends to cause secondary damage that aggravates the patient's condition. The main treatment methods for severe brainstem hemorrhage are currently relieving hematoma compression and inflammatory stimulation as soon as possible, maintaining stable vital signs, and reducing the occurrence of secondary damage. After minimally invasive puncture treatment, urokinase injection is often needed to improve the hematoma drainage. The location of the drainage tube is a key factor in determining the drainage of the hematoma. Laser combined with the XperCT technology for assisting brainstem hematoma puncture and drainage can facilitate accurate placement of the hematoma cavity drainage tube to achieve rapid drainage of the hematoma. At the same time, it has the advantages of low trauma, high accuracy, short operation time, and low operation cost, making it convenient for general application.

## Data Availability Statement

The original contributions presented in the study are included in the article/supplementary material, further inquiries can be directed to the corresponding author.

## Ethics Statement

Written informed consent was obtained from the individual(s) for the publication of any potentially identifiable images or data included in this article.

## Author Contributions

QWa drafted the manuscript and responsible for the revision and data processing of the article. TZ, SW, CL, and ZY completed the operation. WG, QWe, and XG performed the data collection and data analysis. ZL participated in the design of this study and helped to check the manuscript. All authors read and approved the final manuscript. All authors have contributed to the article and approved the submitted version.

## Funding

This study was financially supported by Natural Science Foundation of Shandong (No. ZR2018LH007).

## Conflict of Interest

The authors declare that the research was conducted in the absence of any commercial or financial relationships that could be construed as a potential conflict of interest.

## Publisher's Note

All claims expressed in this article are solely those of the authors and do not necessarily represent those of their affiliated organizations, or those of the publisher, the editors and the reviewers. Any product that may be evaluated in this article, or claim that may be made by its manufacturer, is not guaranteed or endorsed by the publisher.
